# Supplementary material for: Hormonal Defects Are Common during Puumala Hantavirus Infection and Associate with Disease Severity and Biomarkers of Altered Haemostasis
Source: Viruses. 2021 Sep 13;13(9):1818. doi: 10.3390/v13091818 (PMC8472102; doi:10.3390/v13091818)
Supplement: Supplementary file 1 [file viruses-13-01818-s001.zip › Viruses_Supplementary data_ Hormone analysis methods S2.pdf]

Hormone analysis methods

Plasma free thyroxine (fT4), thyrotropin (TSH) and antibodies against thyroid peroxidase (TPOAb) and serum estradiol (E2), follicle-stimulating hormone (FSH), luteinizing hormone (LH), prolactin (PRL) and cortisol were analyzed by electro-chemiluminescence immunoassay (ECLIA) (Elecsys and Cobas e immunoassay analyzers, Roche Diagnostics, Germany). Serum testosterone (Testo) was analyzed by liquid chromatography-mass spectrometry (Agilent 1200 Series RRLC, Agilent Technologies, Germany for chromatography and Agilent 6410 Triple Quadrupole LC/MS (G6410A), Agilent Technologies, USA for mass spectrometry). Serum levels of growth hormone (GH) were measured by a time-resolved immunofluorometric assay (AutoDELFIA, Perkin Elmer, Wallac, Finland), an immunochemiluminometric assay (ILMA) (Immulite 2000Xpi, Siemens, Healthcare Diagnostics, USA) and ECLIA (Cobas 8000, Roche Diagnostics, Germany). Plasma levels of adrenocorticotropic hormone (ACTH) were analyzed by ILMA (Immulite 2000XPi, Siemens, Healthcare Diagnostics, USA) and a chemiluminescence immunoassay (CLIA) (LIAISON XL, DiaSorin, Italy and Immulite 2000, Siemens, Healthcare Diagnostics, USA). Serum insulin-like growth factor-1 (IGF-1) levels were analyzed by ILMA (Immulite 2000 XPi, Siemens, Healthcare Diagnostics, USA) and CLIA (IDS-iSYS, Immunodiagnostics Systems, UK).
